# Inferring replication states of bacteria and viruses in enrichment cultures via long-read sequencing

**DOI:** 10.1093/ismeco/ycaf041

**Published:** 2025-03-05

**Authors:** Sophie A Simon, André R Soares, Till L V Bornemann, Adrian Lange, Lea Griesdorn, Adrián Fuentes, Marie Dieckmann, Beate A Krok, S Emil Ruff, Michael Hügler, Cristina Moraru, Alexander J Probst

**Affiliations:** Environmental Metagenomics, Faculty of Chemistry, Research Center One Health Ruhr of the University Alliance Ruhr, University Duisburg-Essen, Universitätsstraße 5, Essen 45141, Germany; Environmental Metagenomics, Faculty of Chemistry, Research Center One Health Ruhr of the University Alliance Ruhr, University Duisburg-Essen, Universitätsstraße 5, Essen 45141, Germany; Centre for Water and Environmental Research (ZWU), University of Duisburg-Essen, Universitätsstraße 5, Essen 45141, Germany; Environmental Metagenomics, Faculty of Chemistry, Research Center One Health Ruhr of the University Alliance Ruhr, University Duisburg-Essen, Universitätsstraße 5, Essen 45141, Germany; Centre for Water and Environmental Research (ZWU), University of Duisburg-Essen, Universitätsstraße 5, Essen 45141, Germany; Environmental Metagenomics, Faculty of Chemistry, Research Center One Health Ruhr of the University Alliance Ruhr, University Duisburg-Essen, Universitätsstraße 5, Essen 45141, Germany; Environmental Metagenomics, Faculty of Chemistry, Research Center One Health Ruhr of the University Alliance Ruhr, University Duisburg-Essen, Universitätsstraße 5, Essen 45141, Germany; Environmental Metagenomics, Faculty of Chemistry, Research Center One Health Ruhr of the University Alliance Ruhr, University Duisburg-Essen, Universitätsstraße 5, Essen 45141, Germany; Environmental Metagenomics, Faculty of Chemistry, Research Center One Health Ruhr of the University Alliance Ruhr, University Duisburg-Essen, Universitätsstraße 5, Essen 45141, Germany; Centre for Water and Environmental Research (ZWU), University of Duisburg-Essen, Universitätsstraße 5, Essen 45141, Germany; The Marine Biological Laboratory, Woods Hole, MA, United States; TZW: DVGW-Technologiezentrum Wasser, Karlsruhe 76139, Germany; Environmental Metagenomics, Faculty of Chemistry, Research Center One Health Ruhr of the University Alliance Ruhr, University Duisburg-Essen, Universitätsstraße 5, Essen 45141, Germany; Environmental Metagenomics, Faculty of Chemistry, Research Center One Health Ruhr of the University Alliance Ruhr, University Duisburg-Essen, Universitätsstraße 5, Essen 45141, Germany; Centre for Water and Environmental Research (ZWU), University of Duisburg-Essen, Universitätsstraße 5, Essen 45141, Germany; Center of Medical Biotechnology (ZMB), University of Duisburg-Essen, Universitätsstaße 2, Essen 45141, Germany

**Keywords:** nanopore sequencing, genome-resolved metagenomics, 5-bromo-2′-deoxyuridine (BrdU), prokaryotic replication, prophage induction, enrichment cultures

## Abstract

Most microorganisms cannot be cultured in isolation, necessitating sophisticated methods for studying their (eco)physiology. While numerous approaches can probe the activity of given microbes in enrichment cultures, no single technique can render simultaneous data on both metabolic capacities and mobile genetic elements. Here, we apply long-read sequencing to monitor the incorporation of non-canonical bases in genome-resolved metagenomic datasets and elucidate the replication patterns of both bacteria and phages. This technology enables the simultaneous reconstruction of both prokaryotic and viral genomes (alongside genomics downstream analyses like metabolic predictions), in addition to providing information regarding their replication in enrichment cultures. By spiking the base analog 5-bromo-2′-deoxyuridine (BrdU) into activated sludge microcosms, we determined that 114 of the 118 high-quality genomes recovered were actively replicating in enrichment cultures from activated sludge and identified both slow (low BrdU incorporation and change in abundance) and rapidly replicating organisms (high BrdU incorporation and change in abundance). Some of the genomes detected exhibited regions rich in BrdU that were predicted to represent prophages in their lytic cycle. Ultimately, this novel means of monitoring the replication responses of microbes, and deciphering their genomes and active mobile genetic elements will advance and empower strategies aimed at isolating previously uncultivated microbes in pure culture.

As most microbes are refractory to isolation, researchers often study them in ecosystems or enrichment cultures, the latter affording approaches to probe nutrient turnover and/or metabolic activity [1–3]. However, each of these methods has its limitations. To the best of our knowledge, no single technique can be used to simultaneously predict the replication states of microorganisms and mobile genetic elements (e.g. viruses, plasmids) as well as perform genome-resolved metagenomics of enrichment cultures. Methods predicated on genome coverage have proven to be inaccurate, most likely due to lacking consideration for strain heterogeneity [4, 5]. The thymidine base analog 5-bromo-2′-deoxyuridine (BrdU) incorporates into newly synthesized DNA and can be probed with antibodies [6]. While this technology gained popularity in the early 2000s, its application has remained limited to FISH-based microscopic analyses or 16S rRNA gene sequencing [7, 8]. Here, we exploit BrdU to monitor the replication of microorganisms and viruses in each of eight enrichment cultures from activated sludge (four with BrdU amendment and four as a negative control). We monitored these cultures across seven time points, resulting in 56 distinct ONT (Oxford Nanopore Technologies) metagenomes and 15 Illumina libraries (Fig. 1A).

**Figure 1:**
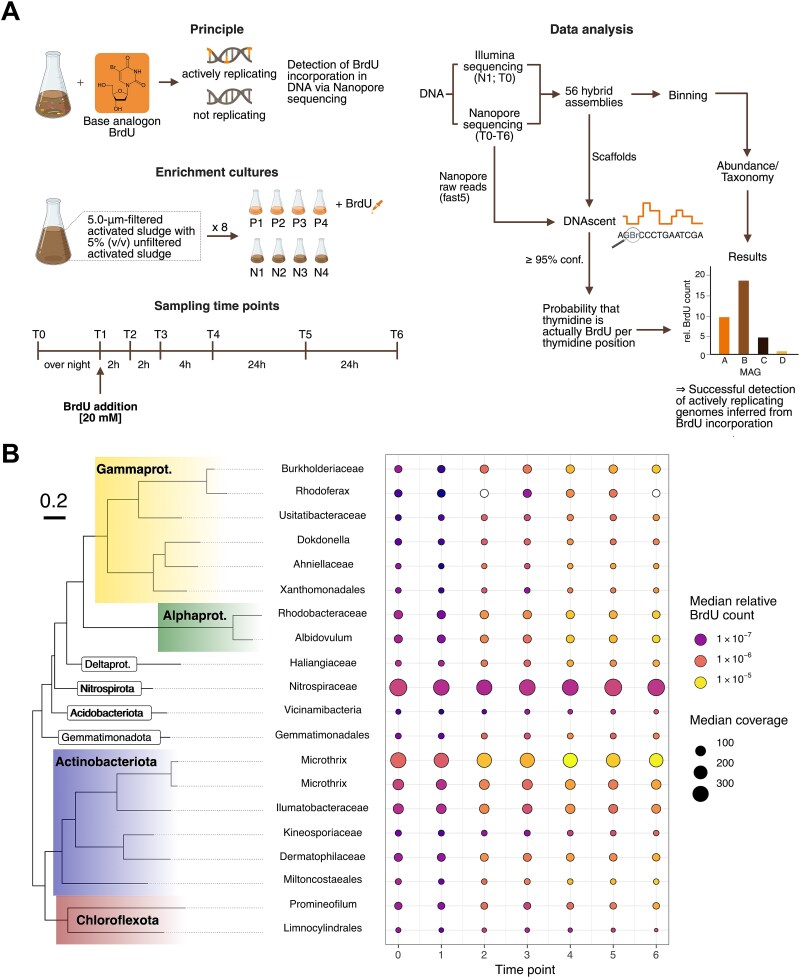
**Experimental design and BrdU incorporation patterns of 20 most abundant organisms.** (**A**) Experimental design of incubations with and without BrdU and respective data analysis workflow. Activated sludge was incubated with and without BrdU for a total of 56 h, during which samples were collected at seven distinct time points. Illumina short-reads and Nanopore long-reads were generated and hybrid-assembled into dereplicated MAGs, whose differential coverage was calculated across all samples. BrdU substitutions were called on long-reads using DNAscent [9] and used to calculate scaffold- and genome-level BrdU containment. (**B**) GTDB phylogeny (tree, colored branch labels) and taxonomic affiliation at phylum- and class-level of the 20 most abundant MAGs according to the median values for genome-level normalized BrdU accumulation across replicates. Labels are provided for each MAG given their lowest known taxonomic rank (y-axis of bubble plot). Median normalized (see **Supplementary Methods—BrdU count normalization**) relative BrdU counts across replicates (bubble color) and coverage (bubble size) of MAGs across time points (x-axis) are displayed in a bubble plot.

We used DNAscent [9], which jointly evaluates mappings of Nanopore reads to a reference (metagenome-assembled genome (MAG) or assembly) and electric signal data of the mapped Nanopore reads, to estimate the probability that a predicted thymidine is more likely BrdU and applied it to complex enrichment cultures from activated sludge (BrdU confidence > = 95%; Fig. 1A). We verified this approach on (i) labeled and unlabeled 16S rRNA gene PCR products and (ii) four pure cultures of easy-to-cultivate model organisms (*Escherichia coli, Bacillus subtilis, Corynebacterium glutamicum,* and *Salmonella enterica* grown in pure cultures spiked with 20 μM BrdU) with well-characterized genomes (BrdU calling confidence > = 90%; see **Methods** in **Supplementary Information** and Supplementary Fig. S1). PCR products generated with either dTTP (unlabeled) or BrdUTP (labeled) were used as control to ensure the exclusive incorporation of BrdUTP in the positive control and the absence of DNA-modifications in the negative control. Long-read sequence metagenomes were then generated from activated sludge enrichments with and without the addition of BrdU, which were tracked over seven time points (Fig. 1A) in sets of four biological replicates each. This sequencing effort was complemented with Illumina short-read sequencing, generating a total of 118 dereplicated high-quality MAGs affiliated with Proteobacteria (38 MAGs), Actinobacteriota (25), Bacteroidota (20), and Chloroflexota (12). Of the 118 total MAGs generated, 114 incorporated BrdU, suggesting a broad applicability of BrdU for enrichment studies. Twenty-nine MAGs spanning nine phyla showed more than 0.001 normalized BrdU counts (see **Methods** in **Supplementary Information**). These included species of *Microthrix* (Actinobacteriota) and several Alpha- and Gammaproteobacterial genera, which were only moderately abundant in the enrichment culture (Fig. 1B). Highly abundant Nitrospirae MAGs showed low BrdU incorporation, which was consistent with their slow growth rate [10] and relative abundance change over time (Fig. 1B). Analyzing the BrdU incorporation in one distinct MAG revealed that BrdU can be incorporated randomly (Fig. 2A), as the origin of replication (*oriC*) did not exhibit significantly increased BrdU detection (Chi-square test, no significant *P*-values (<0.005) detected under the null hypothesis, gray dashed line) and certain regions showed no incorporation at all. MAG-level BrdU incorporation and coverage in samples augmented with BrdU yielded marginal positive correlations (Kendall’s τ = 0.205, *P* < 0.001, with timepoints 0 and 1 excluded as these did not contain BrdU), with minimal false positive BrdU hits detected in samples *sans* BrdU addition (see Supplementary Fig. S3), which are virtually eliminated with the use of R10.4.1 flowcells as demonstrated by BrdUTP PCR (see Supplementary Fig. S4). The yet-to-be improved BrdU calling confidence in combination with metagenomic relative abundance estimations do not yet allow for absolute quantitative insights. Improvement of the BrdU calling confidence with R10.4.1 and future chemistries will also increase the possibility to use the presented BrdU-Nanopore sequencing method in a more quantitative way.

**Figure 2:**
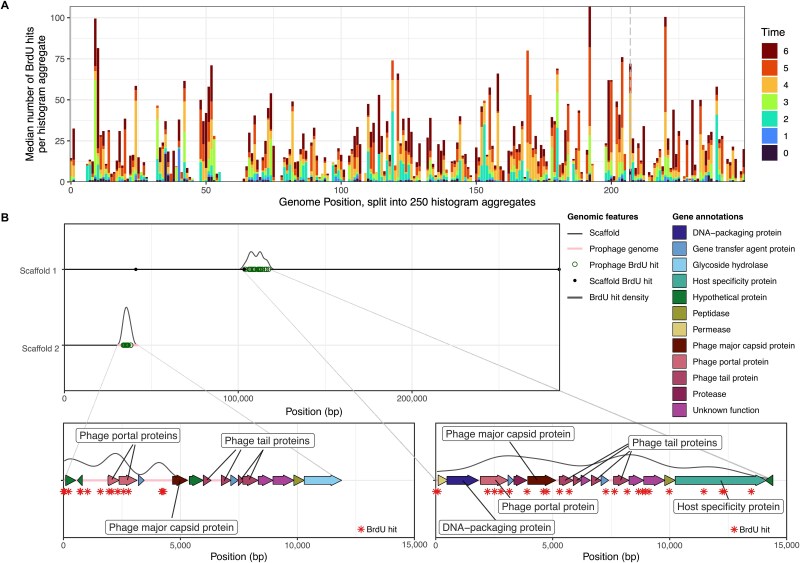
**Examples of distribution of BrdU incorporation across bacterial and prophage genomes.** Genome-wide (x-axis, split into 250 histogram aggregates of ~36 kbp each) BrdU accumulation (bar lengths, y-axis) across time points (bar colors), with vertical dashed line denoting the predicted origin of replication. The displayed MAG is AcBaMe_P2T5_Deltaproteobacteria_bacterium_69_7. (**A**). Visualization of BrdU hits (open circles) across the two prophage- carrying scaffolds (y-axis) found to have the highest differences in BrdU incorporation (filled circles in genomic scaffolds) between the genomic and prophage portion of the scaffold (pink lines below the density curve, **B**). Annotations found for each prophage are expanded across the prophage genomes (x-axis indicates genomic position). Colors and labels indicate annotation, while in the background in gray, a density curve plots the density of BrdU hits (asterisks) across the prophage genomes.

Applying BrdU to a pure culture of *S. enterica* serovar Typhimurium LT2 showed increased BrdU in one of the prophages, making induction by BrdU a possibility (Supplementary Fig. S2). Consequently, focusing on prophages, and using the surrounding scaffold of BrdU incorporation from the host as a baseline, we examined BrdU induction of prophages across the enrichment culture sample sets. Since the same prophages were not induced across replicates (see prophage clustering relative to BrdU incorporation; Supplementary Fig. S7) we conclude that induction observed in some cases was random and not prompted by the addition of BrdU. In these instances, we detected a significant increase in BrdU incorporation in prophage regions encoding for prophage capsid and tail proteins, indicative of phage induction and replication in the hosts’ cytoplasm (Fig. 2B). As such, we recommend the use of BrdU alongside ONT for monitoring prophage induction in respective assays.

Despite the challenges and shortcomings involved in reproducing enrichment cultures, assaying a broad spectrum of substrates and monitoring the growth responses of microorganisms of interest via the herein introduced BrdU-ONT assay will empower efforts toward cultivating not-yet-cultivated microbes. Identifying the induction of prophages that might hinder the growth of uncultivated organisms of interest will factor largely in future strategies to optimize cultivation conditions. In such cultivation efforts, it will be essential to account for the potential cytotoxicity of BrdU which could unintentionally impact microbial growth, yet the concentration of BrdU used in within this study (20 μM) has reportedly shown low toxicity [7]. We believe that as ONTs continue to improve chemistry and flow cell technology (e.g. DNAscent was recently adapted to work with R10 flow cells), activity-based metagenomics will no longer require short-read data to infer growth/replication from high-quality ONT-based MAGs [11]. Taken together, BrdU coupled to ONT base calling is a valuable tool for studying the activity of microbes in enrichment cultures along with deciphering their genomic content.

## Supplementary Material

Simon_et_al_BrdU_SuppText_ISMEComms_final_ycaf041(1)

Simon_et_al_BrdU_ISMEComms_SupplementaryTables_ycaf041

## Data Availability

Raw sequencing data and MAGs have been deposited at SRA and are available under the BioProject PRJNA1156938. Individual BioSample IDs of all MAGs are listed in Supplementary Table S8.
